# The Effect of the New Jersey Law

**Published:** 1903-05

**Authors:** 


### THE EFFECT OF THE NEW JERSEY LAW.

J. W. Haffer, of Paterson, N. J., was recently graduated from the Ontario Veterinary College, Toronto, Canada. He has opened offices in Fall River, Mass. New Jersey's law bars out all graduates of two-year schools.

